# Association between parent-infant interactions in infancy and disruptive behaviour disorders at age seven: a nested, case–control ALSPAC study

**DOI:** 10.1186/1471-2431-14-223

**Published:** 2014-09-06

**Authors:** Christine Puckering, Clare S Allely, Orla Doolin, David Purves, Alex McConnachie, Paul CD Johnson, Helen Marwick, Jon Heron, Jean Golding, Christopher Gillberg, Philip Wilson

**Affiliations:** Institute of Health and Wellbeing, University of Glasgow, RHSC Yorkhill, Glasgow, Scotland G3 8SJ UK; School of Health Sciences, University of Salford, Allerton Building, Frederick Road, Salford, England M6 6PU UK; Robertson Centre for Biostatistics, University of Glasgow, Boyd Orr Building, Glasgow, Scotland G12 8QQ UK; National Centre for Autism Studies, the University of Strathclyde, Scotland, UK; Centre for Child and Adolescent Health, School of Social and Community Medicine, University of Bristol, Bristol, England, UK; Centre for Mental Health, Addiction and Suicide Research, School of Social and Community Medicine, University of Bristol, Bristol, England, UK; Centre for Rural Health, University of Aberdeen, The Centre for Health Science, Old Perth Road, Inverness, Scotland, IV2 3JH UK

**Keywords:** ALSPAC, Disruptive behaviour disorders, Parent-infant interactions, Mellow Parenting Observation System

## Abstract

**Background:**

Effective early intervention to prevent oppositional/conduct disorders requires early identification of children at risk. Patterns of parent-child interaction may predict oppositional/conduct disorders but large community-based prospective studies are needed to evaluate this possibility.

**Methods:**

We sought to examine whether the Mellow Parenting Observational System (MPOS) used to assess parent-infant interactions at one year was associated with psychopathology at age 7. The MPOS assesses positive and negative interactions between parent and child. It examines six dimensions: anticipation of child’s needs, responsiveness, autonomy, cooperation, containment of child distress, and control/conflict; these are summed to produce measures of total positive and negative interactions. We examined videos from the Avon Longitudinal Study of Parents and Children (ALSPAC) sub-cohort who attended the ‘Children in Focus’ clinic at one year of age. Our sample comprised 180 videos of parent-infant interaction: 60 from infants who received a psychiatric diagnostic categorisation at seven years and 120 randomly selected controls who were group-matched on sex.

**Results:**

A negative association between positive interactions and oppositional/conduct disorders was found. With the exception of pervasive developmental disorders (autism), an increase of one positive interaction per minute predicted a 15% (95% CI: 4% to 26%) reduction in the odds of the infant being case diagnosed. There was no statistically significant relationship between negative parenting interactions and oppositional/conduct disorders, although negative interactions were rarely observed in this setting.

**Conclusions:**

The Mellow Parenting Observation System, specifically low scores for positive parenting interactions (such as Responsiveness which encompasses parental warmth towards the infant), predicted later psychiatric diagnostic categorisation of oppositional/conduct disorders.

**Electronic supplementary material:**

The online version of this article (doi:10.1186/1471-2431-14-223) contains supplementary material, which is available to authorized users.

## Background

Conduct disorder (CD), oppositional-defiant disorder (ODD), disruptive behaviour disorder NOS (DBD-NOS) and Attention-Deficit/Hyperactivity Disorder (ADHD) grouped together here as disruptive behaviour disorders, are characterised by a set of externalising disruptive behaviours that occur during childhood. ODD involves repeated negativistic, defiant, disobedient and hostile behaviour toward authority figures. ADHD is characterised by developmentally inappropriate inattention, motor activity and impulsive behaviours which cause impairments in both social and academic functioning. ADHD is a chronic debilitating condition associated with significant costs to patients, families as well as society, specifically social and health care services [1]. CD involves a number of problematic behaviours including oppositional and defiant behaviours and antisocial activities (e.g., lying, stealing, running away and physical violence).

CD has substantial health and social costs and there is an increasingly strong case for screening in early childhood [2]. Without intervention, levels of physical and psychiatric mortality and morbidity are high [3]. In an offender cohort followed up between 1^st^ January 1988 to 31^st^ December 1999, young males were nine times more likely and females 40 times more likely to die compared to young people in the general population [3]. CD is also associated with increased risk of criminality [4]. Early intervention with parents can prevent its development [5] and treatment in early childhood is relatively successful [6], while less success is found with adolescents [7]. About 40% of children with CD will go on to develop antisocial personality disorder [8]. Prediction of risk on the basis of demographic information is unlikely to be sufficiently sensitive or specific [9] and so observational assessment of social interactions, whether by parents or independent observers, may prove useful in early identification.

There is a substantial body of work investigating negative aspects of parenting. For example, low maternal responsiveness during the first year of life is associated with later onset of child disruptive behaviours [10, 11]. During the infant’s first year, exposure to maternal depression has been found to be related to reports of child internalising and externalising problems by the mother in the early school years (6-8 years) [12]. Positive aspects of parenting, such as warmth, positive involvement and secure child-parent attachment may independently affect the risk of developing disruptive behaviour disorders [13, 14]. Lower levels of externalising behaviour in childhood have been found in those children of mothers who displayed significantly higher levels of positive parenting throughout toddlerhood [15].

Given the evidence for the benefit of early interventions, primary care clinicians might benefit from the availability of measures which could assist in the prediction of developmental disorders. The present study, based on a large cohort of infants from the Avon Longitudinal Study of Parents and Children (ALSPAC), investigated whether assessment of parenting behaviours at one year can predict psychopathology at age seven. We examined the utility of both positive and negative parenting behaviours towards infants in predicting the later onset of psychopathologies.

## Methods

### Participants

The sample comprised participants from the Avon Longitudinal Study of Parents and Children (ALSPAC). ALSPAC is an ongoing population-based study investigating a wide range of environmental and other influences on the health and development of children. Pregnant women resident in the former Avon Health Authority in south-west England, having an estimated date of delivery between 1 April 1991 and 31 December 1992 were invited to take part, resulting in a ‘core’ cohort of 13,988 singletons/twins alive at 12 months of age [16]. The study website contains details of all the data that are available through a fully searchable data dictionary (http://www.bris.ac.uk/alspac/researchers/data-access/data-dictionary/).

Ethical approval for the study was obtained from the ALSPAC Law and Ethics Committee and the Local Research Ethics Committees. All adult participants gave their informed consent prior to their inclusion in the study. A 10% sample of the ALSPAC cohort, known as the Children in Focus (CiF) group, attended clinics at the University of Bristol at various time intervals between 4 to 61 months of age. For the current study a sample was drawn from this sub sample of the core ALSPAC cohort of 1240 families (usually mother/infant dyads) who attended the ‘Children in Focus’ clinics when children were 12 months old. A range of measures was collected at the clinic including anthropometry, cognitive function, vision, speech and hearing. At the age of 12 months one of the sessions involved the Thorpe Interaction Measure (TIM) [17]. The TIM involves a carer (usually the mother) and her child looking at a picture book. Adults were asked to engage their child in this activity in the same way they would at home, stopping when the child lost interest. All interactions took place in the same ‘living room’ style environment in the clinics and were videotaped. The video recording was terminated if the child became distressed.

Sixty of these infants were later diagnosed with probable autism, conduct disorder, ADHD, anxiety or depression using the Development and Wellbeing Assessment (DAWBA) [18] which was included in a questionnaire sent to the parents of all children remaining in the cohort at 91 months (7.6 years) of age. In parallel, and completely independently the child’s teacher was asked to complete a questionnaire which included similar information. The DAWBA is a structured diagnostic assessment which relies on parental report as well as teacher reports, but final diagnoses are assigned by a child psychiatrist. More than one psychiatric diagnosis can be assigned, although pervasive development disorder (autism) precludes additional diagnoses. Numerous studies have shown the reliability of DAWBA expert diagnoses to be very satisfactory (i.e. [19, 20],). From the remaining non-case videos, 120 controls, group matched on gender, were randomly selected by the ALSPAC team. For this study we included 160 of these videos where the mother was identified as the lead caregiver; 54 cases and 106 controls. Including just the mothers removed the potentially confounding issue of which parent was present in the videos. Given that fathers were such a small number, conducting separate analysis for each parent would be statistically uninformative.

Wolke et al. [21] investigated whether attrition from the ALSPAC study was systematic or random. They found systematic participant drop-out according to the family variables (having a mother who was single; had no educational qualifications; financial difficulty experiences; being raised in large family where the mother smoked; had a poor relationship with the partner; lived in poor housing; had been involved in crime and been convicted or suffered psychopathology during pregnancy). Attrition did not however alter the association between family factors obtained in pregnancy and disruptive behaviour disorder at 7 years of age and we believe it unlikely that the direction of the associations we have investigated would be modified by the attrition.

### Procedure

The Mellow Parenting Observational System (MPOS) [22] was used to assess parent-infant interactions at one year (see Additional file 1). The observers were blind to case or control status. Measurements of the rate of positive interactions were moderately reliable with an inter-class correlation of 53%. Measurements of the rate of negative interactions had a correlation of 0.60 using Kendall’s τ [23]. Inter-rater reliability for the rate of total positive interactions was assessed using the interclass correlation coefficient. Due to non-normal distribution of the rate of negative interactions, we used Kendall’s τ to assess the agreement between raters. Kendall’s τ gauges concordance among the ranks, not the measures themselves, but we justify its use on the grounds that non-parametric measures of true reliability (i.e. concordance) are not available. Measures with τ > 0.6 were considered reliable. MPOS has previously been shown to have good inter-rater reliability [24]. Within the MPOS, the rater counts the number of occurrences of positive and negative interactions between parent and child. There are six Mellow Parenting dimensions, each rated for positive and negative interactions; Anticipation of Child’s Need; Autonomy; Control and Conflict; Cooperation; Distress; Responsiveness. The positive and negative poles of each dimension were summed to provide total positive and total negative interaction scores.

### Statistical methods

All 160 subjects had available interaction scores and were used in the analyses. Total positive and negative interaction scores were analysed as counts per minute of video material. We also examined whether video duration was associated with diagnostic outcome as some videos were stopped if the infant became distressed.

**Figure 1:**
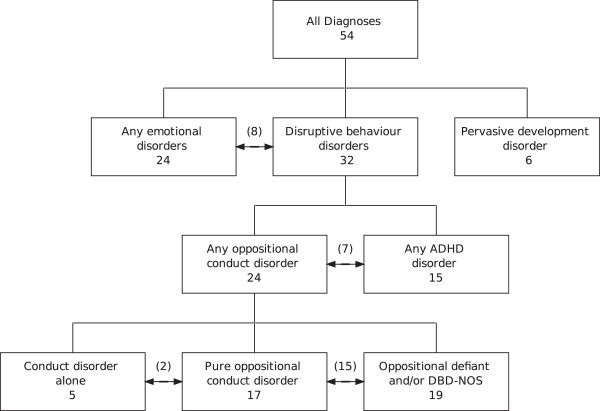
**Structure of psychopathology diagnoses.** The numbers in parenthesis indicate the count of comorbidities present (for example, eight infants diagnosed with both any emotional disorder and disruptive behaviour disorder).

The interaction scores were used in predictive models of case and control status overall and within the following pre-defined sub-diagnostic groups; any ADHD; any emotional disorder; pervasive development disorder; disruptive behaviour disorder (DBD); any oppositional conduct disorder; conduct disorder alone; oppositional defiant and/or DBD-NOS; pure oppositional conduct disorder (Figure 1). Associations between interaction scores and psychiatric disorders were analysed using Firth’s penalized–likelihood logistic regression to correct for biases that may be induced by the low prevalence for some disorders [25]. To account for any potential confounding all models were adjusted for select parental and infant characteristics previously found to be associated with parent/infant interaction scores [24]. When positive interactions were the variable of interest the models were adjusted for the child’s gender, maternal age at birth, maternal educational attainment and pre-natal anxiety scores. Models with negative interaction scores were adjusted for maternal age at birth, maternal smoking status, the child’s gender and a social support score. Odds ratios (ORs), 95% confidence intervals and p-values are presented, with ORs reporting the effect associated with an increase of one interaction count per minute in the respective scores.

All analyses were carried out using R statistical package v2.15.

## Results

The mean duration for the 160 videos used was 211 seconds (SD 86), with a range from 60 to 510 seconds. Table 1 summarises the number of cases overall and within sub-diagnostic groups, with low prevalence noted for pervasive development (autism) and conduct disorder.

**Table 1:** **Summary of the number of children within each diagnostic subgroup (see Figure** 1 **) by gender**

Diagnostic outcome	Total	Gender	
		Female	Male
N_OBS_	160	49	111
N_CASE_	54	16	38
**N (%) of cases**			
Any ADHD disorder	15 (25%)	1 (6%)	14 (34%)
Pervasive development disorder	6 (10%)	1 (6%)	5 (12%)
Any emotional disorder	24 (41%)	11 (61%)	13 (32%)
Disruptive behaviour disorders	32 (54%)	6 (33%)	26 (63%)
Any oppositional-conduct disorder	24 (41%)	5 (28%)	19 (46%)
Conduct disorder alone	5 (8%)	1 (6%)	4 (10%)
Oppositional defiant and/or DBD NOS	19 (32%)	4 (22%)	15 (37%)
Pure oppositional conduct disorder	17 (29%)	5 (28%)	12 (29%)

Video duration was not found to be predictive of diagnostic outcome. The mean (sd) number of positive interactions per minute was 6.2 (3.3) and 0.37 (0.8) for negative interactions. Table 2 presents the associations between diagnostic outcomes and a one count per minute increase in total positive or total negative interactions; there is a trend of increasing positive interactions predicting a reduction in psychopathology diagnoses, including overall diagnosis and across the behavioural subgroups, with the exception of autism, An increase of one positive interaction per minute predicted a 15% (95% CI: 4% to 26%) reduction in the odds of the infant being case diagnosed - as prevalence is low in the wider population the OR can be interpreted as a risk ratio; If the rates of positive interactions increase from 3.7 to 8.0 per minute – from the lower to the upper quartiles of the range – this predicts a 50% (16% to 72%) reduction in the risk of an infant being diagnosed a case Total negative scores were not significantly associated with either overall caseness or any case subgroup although this may partly be explained by the large number of videos (103; 64%) having no negative interactions recorded.

**Table 2:** **Association between interaction scores and the odds of an infant being a case (any diagnosis or each subgroup diagnosis)**

Diagnostic outcome	Total positive interactions		Total negative interactions	
	OR (95% CI)	p-value	OR (95% CI)	p-value
Any diagnosis	**0.85 (0.74, 0.96)**	**0.007**	0.98 (0.60, 1.51)	0.941
Pervasive development disorder	0.98 (0.73, 1.21)	0.864	0.02 (0.00, 1.41)	0.099
Any emotional disorder	**0.82 (0.66, 0.98)**	**0.029**	0.71 (0.21, 1.55)	0.446
Disruptive behaviour disorders	**0.84 (0.71, 0.97)**	**0.020**	1.16 (0.70, 1.82)	0.539
Any ADHD disorder	0.87 (0.69, 1.05)	0.159	1.26 (0.65, 2.18)	0.453
Any oppositional-conduct disorder	**0.81 (0.65, 0.97)**	**0.021**	0.96 (0.45, 1.64)	0.902
Conduct disorder alone	0.74 (0.40, 1.09)	0.166	1.07 (0.10, 2.46)	0.909
Oppositional defiant and/or DBD NOS	**0.81 (0.64, 0.99)**	**0.035**	1.02 (0.45, 1.77)	0.959
Pure oppositional conduct disorder	**0.78 (0.60, 0.97)**	**0.024**	1.14 (0.55, 1.95)	0.678

Models are adjusted for potential confounders (total positive interactions adjusted for child’s gender, maternal age at birth, maternal educational attainment and pre-natal anxiety scores; total negative interactions adjusted for maternal age at birth, maternal smoking status, the child’s gender and a social support score) as described in methods. Odds ratio (OR) estimates are presented for a one count per minute increase in each interaction predictor.

Statistically significant associations (p<0.05) are highlighted in **bold** text.

From the data set available from ALSPAC, a reduced group of twenty predictor variables was selected, by investigator consensus, on the basis of previous literature and face validity. These included parental and infant characteristics, indicators of parental socio-economic status (SES) and maternal pre- and post-natal emotional state (Table 3).

**Table 3:** **Univariate associations of predictors with the rate of positive and negative interaction scores**

		Summary statistics for predictor*	Associations with rate of negative interactions	Associations with rate of positive interactions
Child gender	Female	49 (30.6%)	-	-
	Male	111 (69.4%)	1.71 (0.81, 3.62), p = 0.160	0.89 (0.74, 1.06), p = 0.202
Mother age at birth (for 1 year increase)		29.5 (4.5)	**0.90 (0.83, 0.97), p = 0.004**	**1.02 (1.00, 1.04), p = 0.033**
Parity (per unit increase)		0.7 (0.8)	0.87 (0.56, 1.36), p = 0.550	0.97 (0.88, 1.08), p = 0.584
Maternal depression at 32--40 weeks (per unit increase)		6.9 (5.0)	1.01 (0.94, 1.08), p = 0.812	1.01 (1.00, 1.03), p = 0.118
Postnatal depression at 8 months (per unit increase)		5.6 (5.0)	1.03 (0.97, 1.10), p = 0.354	1.01 (0.99, 1.02), p = 0.478
Maternal anxiety at 32--40 weeks (per unit increase)		4.7 (3.4)	1.03 (0.93, 1.14), p = 0.630	1.02 (0.99, 1.04), p = 0.153
Postnatal anxiety at 8 months (per unit increase)		3.8 (3.9)	1.00 (0.92, 1.10), p = 0.934	1.01 (0.99, 1.04), p = 0.172
Infant breast fed	No	24 (15.1%)	-	-
	Yes	135 (84.9%)	1.26 (0.47, 3.36), p = 0.649	1.19 (0.94, 1.51), p = 0.150
Marital status	Never married	22 (13.8%)	-	-
	1^st^ marriage	123 (77.4%)	1.09 (0.40, 2.97), p = 0.873	1.27 (1.00, 1.63), p = 0.054
	2^nd^/3^rd^ marriage	9 (5.7%)	1.03 (0.18, 5.82), p = 0.970	1.25 (0.82, 1.90), p = 0.292
	Divorced	5 (3.1%)	0.66 (0.07, 6.16), p = 0.718	1.34 (0.80, 2.24), p = 0.264
Father in household	No	14 (9.2%)	-	-
	Yes	139 (90.8%)	0.50 (0.15, 1.63), p = 0.251	1.20 (0.89, 1.62), p = 0.225
Maternal education levels	Vocational/CSE/GCSE	89 (56.0%)	-	-
	A level/Degree	70 (44.0%)	1.02 (0.51, 2.04), p = 0.958	**1.32 (1.12, 1.55), p = 0.001**
Anyone with chronic illness in household	No	133 (88.7%)	-	-
	Yes	17 (11.3%)	1.11 (0.36, 3.42), p = 0.861	0.89 (0.68, 1.16), p = 0.389
Smoked during first trimester	No	128 (81.0%)	-	-
	Yes	30 (19.0%)	0.64 (0.26, 1.58), p = 0.331	0.91 (0.73, 1.13), p = 0.384
Alcohol during first trimester (glasses of alcohol per week)	< 1	129 (81.6%)	-	-
	≥ 1	29 (18.4%)	1.04 (0.43, 2.52), p = 0.929	1.04 (0.84, 1.29), p = 0.737
Partner physically hurt mother at 18 weeks gestation	No	143 (93.5%)	-	-
	Yes	10 (6.5%)	1.29 (0.32, 5.17), p = 0.718	1.03 (0.72, 1.46), p = 0.880
Partner physically hurt mother postnatally	No	152 (95.0%)	-	-
	Yes	8 (5.0%)	0.34 (0.06, 1.97), p = 0.230	1.01 (0.69, 1.47), p = 0.962
Social support score (per unit increase)		20.1 (4.8)	0.94 (0.87, 1.01), p = 0.072	1.01 (0.99, 1.03), p = 0.335
Life event score 18--23 weeks (per unit increase)		8.6 (6.5)	1.02 (0.97, 1.08), p = 0.417	1.00 (0.98, 1.01), p = 0.716
Maternal bonding score (per unit increase)		28.0 (4.0)	0.98 (0.91, 1.07), p = 0.723	**0.98 (0.96, 1.00), p = 0.024**
Aggression score (per unit increase)		10.2 (1.8)	0.96 (0.78, 1.17), p = 0.655	1.03 (0.98, 1.08), p = 0.300

(Effect estimates are the relative change in interaction scores for a specified increase in continuous predictor variables or compared to the stated reference group for categorical predictors).

*Mean (SD) presented for continuous variables and N (%) for categorical.

- indicates reference category in regression analysis.

Statistically significant associations (p<0.05) are highlighted in **bold** text.

Backward stepwise regression analysis revealed three variables to be independent predictors of positive interactions. Higher rates of positive interaction were observed with older mothers, mothers with a higher level of education and mothers who experienced anxiety during the third trimester. Four variables independently predicted the rate of negative interactions. Specifically, there were fewer negative interactions seen in older mothers, mothers who perceived that they received more social support during pregnancy (encompassing perceived emotional and financial support from a partner, friends, family, neighbours, other pregnant women and the state), mothers who smoked during the first trimester and mothers with female infants.

## Discussion

Based on a large community-based cohort of infants, we investigated whether observations of mother-infant interactions at one year analysed using the Mellow Parenting Observational System (MPOS) can predict psychopathology at age seven. A negative association was found between total positive interactions and overall case diagnosis, in disruptive behaviour disorders and in emotional disorders; those with conduct disorder alone had the lowest total positive interaction scores, though with only five cases, the association did not reach statistical significance in this subgroup. There were no significant associations between negative parenting interactions and later diagnoses of psychopathology. This may have been a consequence of the low power due to the low number of negative interactions observed. The relatively low power for detecting associations with negative interactions is reflected in their relatively wide effect estimate confidence intervals (Table 2). The Mellow Parenting Observational System may therefore be capable of assisting the early identification of later development of psychopathology. While the findings support an effect for positive interactions and not for negative interactions, we have been careful not to draw strong conclusions from the various sub-group results. Quality of parenting in early childhood has been reported to predict later conduct problems [26]. Warm interaction and maternal responsiveness may be necessary components for the development of compliance and internalised controls [27]. Limit-setting and discipline may thus not be as effective in the absence of a positive and warm parent-child relationship and may indeed not be salient in infancy. Low maternal responsiveness during the first year of life is associated with later onset of child DBDs [10, 28] and mothers of children with behaviour problems are likely to be less warm [29] and not as involved positively [30] when compared with other mothers. Disruptive preschool boys are less likely to have a secure attachment to their mother [31]. We have recently reported that low frequencies of *maternal* vocalisation predicted later development of infant psychopathology in the ALSPAC cohort [32].

The importance of parent-child interactions [33] has been widely emphasised and these interactions are likely to feature in the causal pathways for antisocial behaviour. Assessment of parenting behaviours early in life may therefore shed some light on the association between parenting style and later development of oppositional/conduct disorders.

### Limitations and strengths of the study

The camera recording the mother-infant interaction was placed in the upper corner of the room, so the faces of parents and children were often not visible. The video quality was relatively poor due to the age of the tapes, which may have contributed to the moderate reliability of the MPOS. Given the positive relationship between reliability and sensitivity, we might expect the use of more modern video equipment to substantially improve the sensitivity of the MPOS. Despite such limitations, we *were* able to confirm our hypothesis of an association between parenting behaviours and later development of conduct disorder. Our data have particular value because of their community base: previous studies have sampled high risk referred children or siblings of affected individuals and we have published a number of findings using the same dataset [32, 34–36]. Also of note is that the predictor variables used in our analyses were based on videos recorded at one year of age, which were rated without knowledge of the future psychopathology of the child; the data can therefore be viewed as prospective, in contrast to previous studies which have been based on retrospective recall of predictive factors. There was no statistically significant relationship between negative parenting interactions and any of the diagnostic categories: a potential limitation of the present study is that negative interactions were rarely observed in this setting. We have performed numerous statistical tests, without adjustment for multiple comparisons, so there is the possibility of Type I error; however, overall case diagnoses was pre-defined as the primary outcome with the hierarchy of sub-diagnoses also defined in advance.. Given that significant associations were observed between total positive interactions and several sub-diagnoses, this adds to the robustness of our findings. The setting used for the TIM did not elicit many negative interactions: it was not devised specifically to study the type of negative interactions for which the MPOS is designed. Nevertheless, the MPOS did identify a large number of positive interactions, and substantial variability in these behaviours was demonstrated by the ALSPAC participants. Furthermore, if videos were available of settings specifically designed to elicit a range of positive and negative interactions, then this could enhance the reliability of the MPOS measure. Finally, some diagnoses, for example, pervasive developmental disorder, were represented by small numbers and the lack of associations may reflect Type II error. It is possible that lower positive parenting scores may represent an overall lower level of maternal activity: we have reported that less frequent maternal vocalisations [32] and lower levels of parental activity using a holistic measure [34] are associated with later psychopathology and we are currently exploring the inter-relationships between these predictors.

It was difficult to achieve high reliability on the MPOS in this setting, for which it was not uniquely designed. There are similar problems in applying other possible coding systems, for example the Care Index which has specific instructions based around three minutes of free play, rather than the constrained conditions of the TIM. Nevertheless MPOS has been shown to have utility and predictive or concurrent validity in other situations. For example, it distinguishes parent-child dyads with growth delay [37] and is sensitive to change, concurrently with maternal depressed mood [22]. Though not as well supported by reliability and validity studies as would be ideal, it was therefore chosen as a tool. It effectively predicts DBDs in this study, some six years after the original coding, supporting the assertion that is a useful system, albeit in need of further refinement for future use.

### Clinical implications

There is a need for tools which can be used by primary care clinicians to assist in early identification of disruptive behaviour disorders. While we acknowledge that further investigation of the concurrent and predictive indicators of the MPOS measure is required, these initial results indicate that positive parenting, as measured by the MPOS *may* be useful in assisting in the early detection of risk for disruptive behaviour disorders and it is possible that with further refinement it could be used to assess parent-infant interactions in primary care settings.

### Future research

It is possible that observational assessment may have greater utility in more naturalistic social interactions than those studied here. Further community-based longitudinal studies of the predictive validity of the MPOS in different types of social interaction, for example feeding, nappy changing or free play are indicated. Situations where parenting skills are challenged by the task and thus negative interactions more likely to be observed may have additional value in predicting the onset of disruptive behaviour disorders.

## Conclusions

Despite many investigations of negative aspects of parenting, much less research has focused on the impact of positive parenting processes. It is increasingly recognised that positive aspects of parenting, such as warmth, positive involvement and secure child-parent attachment are independently associated with a reduced risk of developing disruptive behaviour disorders and may be particularly salient in the very early years before behaviour management strategies predominate [13]. The work reported here lends some support to this finding.

## Electronic supplementary material

Additional file 1: **Mellow Parenting Observational System.** (DOC 28 KB)

## Abbreviations

MPOS: Mellow Parenting Observational System
ALSPAC: Avon Longitudinal Study of Parents and Children
CD: Conduct disorder
ODD: Oppositional-defiant disorder
DBD-NOS: Disruptive behaviour disorder NOS
ADHD: Attention deficit hyperactivity disorder.
