# Supplementary material for: Association between parent-infant interactions in infancy and disruptive behaviour disorders at age seven: a nested, case–control ALSPAC study
Source: BMC Pediatr. 2014 Sep 6;14:223. doi: 10.1186/1471-2431-14-223 (PMC4177234; doi:10.1186/1471-2431-14-223)
Supplement: Supplementary file 1 — Additional file 1: Mellow Parenting Observational System. (DOC 28 KB) [file 12887_2014_1157_MOESM1_ESM.doc]

**Additional file 1**

**Mellow Parenting Observational System**

There are six dimensions in the Mellow Parenting Observational System, all recorded in mini-sequences which contain coded behaviours that are positive or negative, and specific to each domain from both the parent and child. Each behavioural event coded represents a single score in the relevant dimension. These events are then summed to give an overall positive and negative score for each dimension. The dimensions are:

**Anticipation of Child’s Needs** – A parent can be seen to prepare the child positively for changes in activity or caretaking by facilitating a known routine, giving prior warning, providing information or distracting the child so that the parent’s agenda is easier to achieve and accomplished with the least possible friction between the dyad. Conversely, negative anticipation occurs when a parent fails appropriately to set up the child for some activity, and then complains to the child or, is negative about the child’s behaviour resulting from the parental lapse.

Examples of Positive Anticipation

• Parent holds out arms to child and waits a moment.

• Parent says “time to go” before picking child up.

Example of Negative Anticipation

• Parent unexpectedly turns on shower over the baby’s head, and infant almost topples over in surprise, followed by parent complaining “Why can’t you sit still?”

**Autonomy** – Parent is seen to show an awareness of the child’s individuality. The child is allowed to exercise choice, to behave spontaneously while the parent monitors ongoing activity. Parent can offer encouragement and help when child is trying things out and heeds child’s protests or complaints. Poor practice occurs where autonomy is not given in these ways where parent is intrusive or child protest is ignored or dealt with in a negative way.

Examples of Positive Autonomy

• Parent holds up a choice of banana and orange in offer to the infant.

• As child is pushing a wagon, parent moves an object which would block it.

Examples of Negative Autonomy

• Child complains and fusses whilst being dressed, and parent continues with no acknowledgement of infant’s distress.

• Child, upset, takes toy from parent’s lap and parent does not respond.

**Responsiveness** – Warmth and stimulation. Parent and child display positive and negative affect to each other in a variety of ways: by tone, demeanour, gesture and verbally. The mutual, positive affective quality such as ‘having fun together’ during caretaking is noted, as is a lack of emotional containment with negativity such as hostile criticism or a lack of support and rough treatment.

Examples of Positive Responsiveness

• Child feeds a toy rabbit, parent responds “The rabbit says yum-yum”.

• Parent and child play peek-a-boo whilst parent dresses infant.

Examples of Negative Responsiveness

• Child is whining and parent responds “I’ve told you to shut up”

• Use of aggressive physical force by either parent or infant, such as smacking.

**Co-operation** – Where the parent and child are each compliant to the other, or are able to negotiate. The parent finds a positive way to influence the child’s behaviour and gain the child’s co-operation. Symmetrical co-operation - “you scratch my back and I’ll scratch yours” - is important. Forcible compliance, threats to achieve compliance and ignored child requests are all negative co-operation.

Example of Positive Co-operation

• Child is asked to leave the television alone and doesn’t, parent says “look, I’ve got your cars here”, child leaves television.

Example of Negative Co-operation

• Parent threatens “If you don’t stop throwing toys I’ll smack you”

**Child Distress** - Where comfort and support is offered to a child who is upset, hurt or miserable. Positive results include resolving the issue causing distress and containing emotional upset. Negatively, parents can precipitate distress and/or fail to support or “mop-up” a distressed child.

Example of Positive Distress

• Child has been upset and parent puts arms around him to give him a close hug.

Example of Negative Distress

• Child gets soap in her eye and cries, parent responds “You would”.

**Control** – Where parent intends to achieve compliance from the child and get them to do what they want Control is coded. Control always starts with non-compliant, oppositional or prohibited child behaviour. Each control issue is judged as legitimate and appropriate for a parent to pursue, and the content of the argument is noted. Whether control issues are nicely and effectively handled, or not, is noted. Compliance or a good solution can be arrived at with or without escalating tension resulting in conflict.

Example of Positive Control

• Child pulls the dog’s tail, parent says “No”, child pulls the dog’s tail again and parent picks up child and distracts child with another activity.

Example of Negative Control

Child is lining up toys while playing, parent says “No, don’t put them on that side, put them over there”, parent proceeds to control how infant plays with the toys.
